# Vitamin D, Gestational Diabetes, and Measures of Glucose Metabolism in a Population-Based Multiethnic Cohort

**DOI:** 10.1155/2018/8939235

**Published:** 2018-04-19

**Authors:** Åse Ruth Eggemoen, Christin Wiegels Waage, Line Sletner, Hanne L. Gulseth, Kåre I. Birkeland, Anne Karen Jenum

**Affiliations:** ^1^Department of General Practice, Institute of Health and Society, University of Oslo, Oslo, Norway; ^2^Department of Child and Adolescence Medicine, Akershus University Hospital, Lørenskog, Norway; ^3^Department of Endocrinology, Morbid Obesity and Preventive Medicine, Oslo University Hospital, Oslo, Norway; ^4^Department of Noncommunicable Diseases, Norwegian Institute of Public Health, Oslo, Norway; ^5^Department of Transplantation Medicine, Oslo University Hospital, Oslo, Norway; ^6^Institute of Clinical Medicine, Faculty of Medicine, University of Oslo, Oslo, Norway; ^7^General Practice Research Unit (AFE), Department of General Practice, Institute of Health and Society, University of Oslo, Oslo, Norway

## Abstract

**Objective:**

We explored associations between maternal 25-hydroxyvitamin D (25(OH)D) status during pregnancy and gestational diabetes (GDM) and other measures of glucose metabolism.

**Methods:**

We analysed 25(OH)D at 15 and 28 gestational weeks (GW) in 745 multiethnic pregnant women attending antenatal care units in Oslo, Norway, between 2008 and 2010. GDM was diagnosed with a 75 g oral glucose tolerance test at 28 GW. Separate regression analyses were performed to investigate associations between 25(OH)D and GDM and measures of glucose metabolism.

**Results:**

A higher proportion of ethnic minority women had GDM (*p* < 0.01) and low 25(OH)D (*p* < 0.01) compared to Europeans. In logistic regression analyses, 25(OH)D < 50 nmol/L was associated with GDM after adjusting for age, parity, education, and season (OR 1.6; 95% CI 1.1–2.2). After additional adjustments for variables reflecting fat mass (skinfolds or BMI) and ethnicity, the association disappeared with ethnicity having a much stronger effect than the adiposity variables. We got similar results exploring effects on other measures of glucose metabolism and when change in 25(OH)D from inclusion to 28 GW was taken into account.

**Conclusions:**

Vitamin D deficiency was not associated with GDM or glucose metabolism in a multiethnic population-based study, after adjustments for confounding factors, in particular ethnicity.

## 1. Introduction

Although the role of vitamin D in calcium metabolism and bone health is undisputed, other long-term health consequences of low vitamin D are still debated [1, 2]. During the last decade, many observational studies have reported an association between low levels of vitamin D and impaired glucose metabolism and type 2 diabetes [1, 3], although results from trials so far have not confirmed a causal relationship [1, 4]. Observational studies have indicated that vitamin D deficiency may be a modifiable risk factor also for gestational diabetes mellitus (GDM) [5], and some studies have found associations with other measures of glucose metabolism in pregnancy [6]. Several reviews and meta-analyses have recently assessed the relation between vitamin D and GDM in observational studies [7–12]. A modest increase in odds of GDM has been found, with a range from 1.38 to 1.61 in women with low levels of vitamin D [5]. Subgroup analyses have found differences based on countries, analytical methods for vitamin D, definition of GDM, maternal age, sample size, adjustment for confounders, and study quality [8], confirming the complexity of interactions among individual, lifestyle, and geographical factors [5].

Vitamin D deficiency in pregnancy is widespread [13], and in Europe, women in ethnic minority groups from South Asia, the Middle East and Africa are at highest risk [14–16]. Possible biological mechanisms of the association between vitamin D and GDM are through effects on insulin-sensitive tissues, calcium pool dysregulation in the pancreas, genetic variations, or inflammation [5, 6]. In addition, *β*-cells may directly convert vitamin D into its active form as both the vitamin D receptor and the 1*α*-hydroxylase enzyme have been found expressed in a pancreatic islet [17]. During pregnancy, irrespective of the prepregnant level, insulin resistance increases about 50–60% [18, 19] but is exaggerated by excessive gestational weight gain. GDM reflects both increased insulin resistance and *β*-cell dysfunction, and both these components have been found to be associated with vitamin D levels [6]. Further, we have previously found a high prevalence of vitamin D deficiency [14], GDM [20] and measures of glucose metabolism, especially in ethnic minority groups, making us capable of exploring relations between vitamin D deficiency, ethnicity, and GDM. Therefore, in the present study, we aimed to assess associations between maternal 25-hydroxyvitamin D (25(OH)D) and GDM and measures of glucose metabolism, insulin resistance, and *β*-cell function, before and after adjusting for potentially confounding factors.

## 2. Materials and Methods

### 2.1. Design, Setting, and Study Population

Data are from a population-based, prospective cohort of 823 presumably healthy women attending maternal and child health clinics for antenatal care in Groruddalen, Oslo, Norway, between May 2008 and March 2010 (the STORK Groruddalen study) [21, 22]. The majority (75–85%) of pregnant women residing in this area, situated at a latitude of 60°N, attended the child health clinics for antenatal care. The study design has been described in detail elsewhere [21, 22]. In short, information material and questionnaires were translated into Arabic, English, Sorani, Somali, Tamil, Turkish, Urdu, and Vietnamese and quality checked by bilingual health professionals. Women were eligible if they (1) lived in the district, (2) planned to give birth at one of the two study hospitals, (3) were in <20 gestational weeks (GW), (4) were not suffering from diseases necessitating intensive hospital follow-up during pregnancy, (5) could communicate in Norwegian or any of the specified languages, and (6) were able to provide written consent. In total, 59% of the included women had an ethnic minority background. The participation rate was 74%, and the participating women were found representative of the main ethnic groups [20]. Maternal data were collected at 15 and 28 GW, through interviews by study personnel, assisted by professional interpreters when needed. Clinical measurements and blood samples were collected according to the study protocol.

### 2.2. Ethics

The Regional Committee for Medical and Health Research Ethics for Southeast Norway (ref. REK 2007/894) and the Norwegian Data Inspectorate approved the study protocol.

Participation was based on informed written consent.

### 2.3. Study Sample

Of the 823 women included in the STORK Groruddalen project at 15 GW, 772 met at 28 GW and 768 of these women had valid data on GDM status by the WHO 2013 criteria. Thirteen women were excluded because of missing data on 25(OH)D at inclusion (15 GW), and ten women from South or Central America were excluded due to low numbers from this geographic region, leaving a study sample of 745 (91%) women (flow chart, Supplementary Figure S1). For secondary outcomes, the sample size was 731 due to some missing values for C-peptide.

### 2.4. Variables

#### 2.4.1. Outcome Variables

The primary outcome in this particular analysis was GDM by the WHO 2013 criteria (fasting plasma glucose (FPG) ≥ 5.1 or 2 h glucose ≥ 8.5 mmol/L) by vitamin D status [20, 23]. We used a modified version of the WHO 2013 criteria as 1-hour PG ≥ 10 mmol/L was not collected. A standard 75 g oral glucose tolerance test was performed at 28 GW [20], and venous blood glucose was measured on-site with a plasma-calibrated HemoCue glucose 201+ (Angelholm, Sweden).

Secondary outcome variables were FPG, 2-hour PG, insulin resistance (measured by homeostasis model assessment of insulin resistance (HOMA-IR)), *β*-cell function (HOMA-B), fasting serum insulin, and C-peptide, all measured at the follow-up visit at 28 GW [19]. C-peptide and insulin were measured at the Hormone Laboratory, Oslo University Hospital, by noncompetitive immunofluorometric assays (DELFIA, PerkinElmer Life Sciences, Wallac Oy, Turku, Finland). HOMA-IR and HOMA-B were estimated by the Oxford University HOMA Calculator 2.2 from the glucose and C-peptide concentrations [24]. Plasma glucose values used in the calculations of a homeostatic model were measured at the Akershus University Hospital from venous blood on gel tubes (VITROS 5,1 FS, Ortho Clinical Diagnostics, slide-adapted colorimetric method).

#### 2.4.2. Main Exposure Variable

Serum 25(OH)D was analysed by competitive RIA (DiaSorin) at the Hormone Laboratory, Oslo University Hospital, at 16 and 28 GW. The method measures total 25(OH)D (both 25(OH)D_2_ and D_3_), with interassay coefficients of variation (CV) of 13–16%. The laboratory is accredited by the International Organization for Standardization and is part of the Vitamin D Quality Assessment Scheme, DEQAS. Concentrations of 25(OH)D < 12 nmol/L were replaced with “11 nmol/L” in the calculations (*n* = 17) so as to not overestimate the effect of low-vitamin D status. The laboratory's reference range was 37–131 nmol/L based on the ethnic Norwegian population from the Oslo Health Study [25]. Preplanned, and according to the protocol, women with 25(OH)D less than the laboratory's lower reference range (<37 nmol/L) at 15 and 28 GW were provided written information about their 25(OH)D concentration and recommended to consult their general practitioner for treatment [14].

#### 2.4.3. Confounders

We performed a search of the literature for relevant confounders, set up a directed acyclic graph (DAG), and selected variables available in our cohort. Maternal age at inclusion was self-reported. Parity was categorised as nulliparous, uniparous, or multiparous (≥2), referring to status before the current pregnancy. Education level was categorised as completed primary education or less (<10 years), completed high school education (10–12 years), and completed ≥4 years college/university education. Season for 25(OH)D measurements at inclusion (15 GW) was categorised as summer (June to November) and winter (December to May) [14]. Specially trained study personnel performed maternal anthropometric measurements at 15 and 28 GW [21]. Each measurement was taken twice, and the mean used. We here report “sum of skinfolds,” which is the sum of the triceps, the subscapular, and the suprailiac skinfold. A change in the sum of skinfolds was calculated as the difference between “sum of skinfolds” at 15 and 28 GW. Ethnic origin was defined by the pregnant participant's mother's country of birth [26]. Ethnic origin was further categorised as Europe (primarily from Norway and Sweden) and ethnic minority women, consisting of South Asia (primarily from Pakistan and Sri Lanka), Middle East including North Africa (primarily Turkey, Iraq, Afghanistan, and Morocco), Sub-Saharan Africa (primarily from Somalia, Eritrea, and Ethiopia), and East Asia (primarily from Vietnam and the Philippines). Prepregnancy BMI was calculated from self-reported weight before pregnancy and height measured at inclusion. Weight gain was calculated as the difference between self-reported prepregnant weight and measured weight at 28 GW. Dietary clusters were derived from four clusters reported earlier [27] and dichotomized as healthy (cluster 4) and unhealthy (clusters 1, 2, and 3).

### 2.5. Statistical Analyses

**Figure 1:**
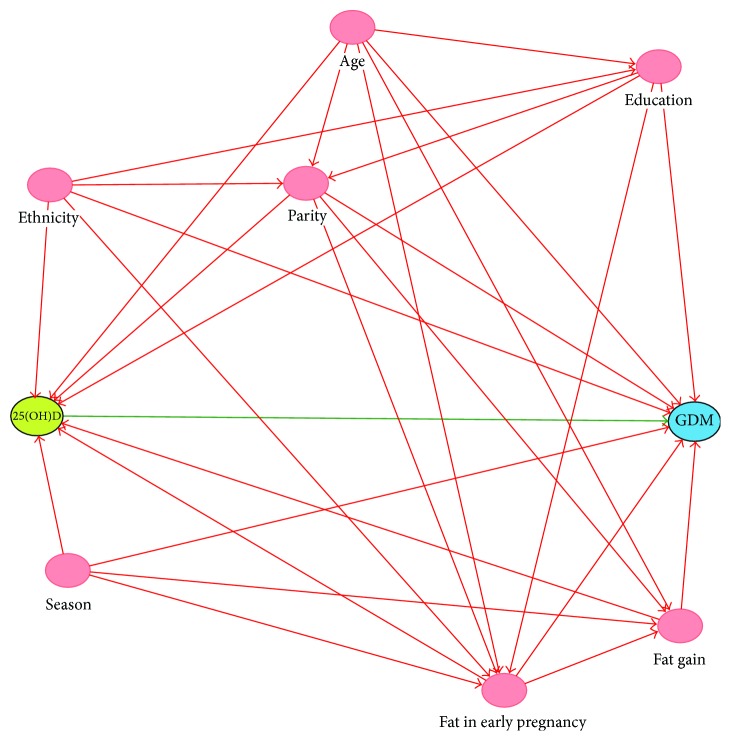
Directed acyclic graph of confounders between vitamin D (25(OH)D) and gestational diabetes mellitus (GDM).

Descriptive statistics are presented as frequencies with proportions, mean values with standard deviations or 95% confidence intervals, and medians with interquartile range. All continuous response variables were assessed for normality. Nonparametric correlation coefficients between 25(OH)D at inclusion and secondary outcomes, assessed at 28 GW, were analysed. Differences between GDM and non-GDM women were tested by *t*-tests for continuous variables and chi-square test or Fischer's exact test for categorical variables. Logistic regression analyses were performed to investigate associations between 25(OH)D and GDM. Separate generalised linear models were performed to assess the relationship between the concentration of 25(OH)D and the secondary outcomes found significant in the correlation analysis (FPG, HOMA-IR, fasting insulin, and C-peptide). Maternal 25(OH)D was analysed as a continuous variable and categorised according to deficiency status (<50 nmol/L or ≥50 nmol/L). As treatment was recommended when 25(OH)D < 37 nmol/L, we further categorised the 25(OH)D status during pregnancy: consistently sufficient level (≥37 nmol/L at 15 and 28 GW), consistently deficient level (<37 nmol/L at 15 and 28 GW), increasing level (<37 nmol/L at 15 GW and ≥37 nmol/L at 28 GW), and decreasing level (≥37 nmol/L at 15 GW and <37 nmol/L at 28 GW). Guided by a DAG (Figure 1), we chose to account for the following *potential* confounders in the regression analyses: age, parity, education, season for measurement of 25(OH)D at 15 GW, sum of skinfolds at 15 GW, change in skinfolds from 15 GW to 28 GW, and ethnicity/geographic origin. Interactions between 25(OH)D and season and between 25(OH)D and ethnicity were examined graphically and by adding interaction terms into the models. We performed sensitivity analysis including prepregnant BMI and weight gain from 15 to 28 GW instead of “sum of skinfolds” at inclusion and change in “sum of skinfolds” to explore the impact of different measures of adiposity. We also included the variable of dietary clusters in the final model as a sensitivity analysis. As insulin, C-peptide, and HOMA-IR values were skewed, these variables were log-transformed, and we repeated the regression analyses with these variables. Results from regression analysis are presented as odds ratio (OR) and coefficients (*β*) with 95% CI. *p* values < 0.05 were considered statistically significant. SPSS software (version 22; IBM SPSS Statistics) and Stata/Se14.1 were used for statistical analysis.

## 3. Results

**Figure 2:**
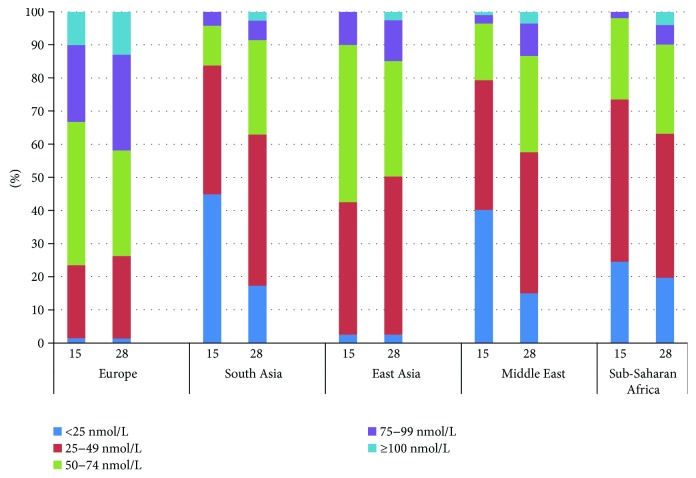
Proportion of participants (%) in categories of serum 25(OH)D concentrations at 15 and 28 weeks of gestation.

**Figure 3:**
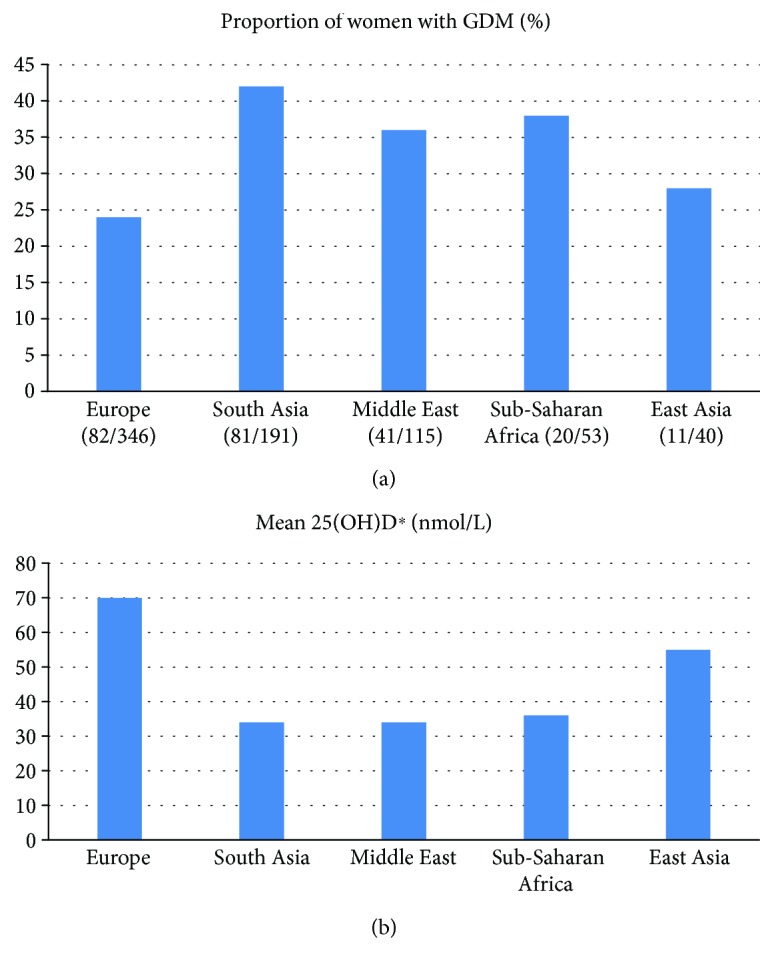
(a) Ethnic variation in gestational diabetes (GDM). (b) Ethnic variation in serum 25(OH)D concentrations. 25(OH)D: 25-hydroxyvitamin D; ^∗^15 gestational week.

**Figure 4:**
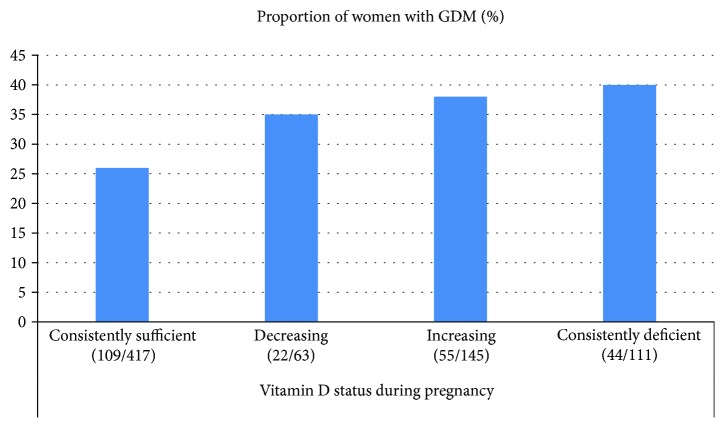
Consistently sufficient: 25(OH)D ≥ 37 nmol/L at 15 and 28 gestational weeks (GW). Decreasing: 25(OH)D ≥ 37 nmol/L at GW 15 and <37 nmol/L at 28 GW. Increasing: 25(OH)D < 37 nmol/L at GW 15 and ≥37 nmol/L at 28 GW. Consistently deficient: 25(OH)D < 37 nmol/L at 15 and 28 GW.

Sample characteristics for the total cohort are presented in Table 1 and stratified by ethnic groups in Supplementary Table 1. Age, sociodemographic and anthropometric variables, the prevalence of GDM, and 25(OH)D levels differed by ethnicity. The concentration of 25(OH)D ranged from <12 to 148 nmol/L at 15 GW, with large ethnic differences; only 35 women from Europe and 1 woman from the Middle East had values > 100 nmol/L (Figure 2). The proportion with vitamin D deficiency (25(OH)D < 50 nmol/L) was significantly higher among women with GDM than among non-GDM women (60% versus 49%, *p* < 0.01) (Table 1). Similarly, the proportion with severe deficiency (25(OH)D < 25 nmol/L) tended to be higher among GDM women (Supplementary Table 2). At 28 GW, the proportion with 25(OH)D < 50 nmol/L was reduced, but differences between GDM and non-GDM women remained significant (Supplementary Table 2). A higher proportion of women with consistent vitamin D deficiency and with levels increasing from low to sufficient were found among GDM women compared to non-GDM women (both *p* < 0.01) (Table 1). A higher proportion of ethnic minority women had GDM (Figure 3(a)) and low 25(OH)D (Figure 3(b)) compared to European women. In addition, a higher proportion of vitamin D-deficient women had GDM compared with women with a consistently sufficient level of vitamin D during pregnancy (Figure 4).

In univariate analyses, vitamin D deficiency (<50 nmol/L) at 15 GW and the categories consistently deficient and increasing of vitamin D status during pregnancy were significantly associated with GDM, but analysed as a continuous variable, 25(OH)D was not significantly associated with GDM (*p* = 0.07). Significant inverse correlations were found between 25(OH)D in early pregnancy and FPG, HOMA-IR, fasting insulin, and fasting C-peptide but not with HOMA-B or 2-hour PG (Supplementary Table 3).

Possible confounders for the relation between 25(OH)D and GDM are presented in Table 1. After adjustments for age, parity, education, and season, vitamin D deficiency was still associated with GDM (model 1; OR 1.6; 95% CI 1.1–2.2) (Table 2). The OR was slightly reduced and the association was no longer significant after additional adjustments for the “sum of skinfolds” and change in the “sum of skinfolds” (model 2). Including ethnicity into the model, the OR was even more attenuated (model 3). Similarly, we found no association with GDM using vitamin D status during pregnancy after adjustments for confounders (Table 2). Based on the correlation analyses, we performed linear regression models for FPG, HOMA-IR, fasting insulin, and C-peptide (Table 3). All significant associations present in unadjusted analyses and model 1 disappeared after adjustments for confounders, with ethnicity having a much stronger effect than the adiposity variables.

### 3.1. Sensitivity Analyses

Using the same approach, but adjusting for prepregnant BMI and weight gain from 15 to 28 GW instead of the “sum of skinfolds” at 15 GW and change in the “sum of skinfolds” in model 2, the association was still significant (results not shown). Including dietary clusters into the models had no effect on the estimates. Using the log-transformed outcome variables in the regression models, we found exactly the same pattern of associations.

## 4. Discussion

In this population-based multiethnic cohort of pregnant women with a high proportion with vitamin D deficiency during the first and second trimesters of pregnancy, we found that the crude prevalence of vitamin D deficiency was higher in women with GDM compared to normoglycemic women. We also found significant inverse correlations between 25(OH)D in early pregnancy and FPG, HOMA-IR, fasting insulin, and fasting C-peptide. However, in fully adjusted regression models, taking into account a number of possible confounders, low levels of 25(OH)D did not predict the development of GDM or deterioration in glucose metabolism observed from 15 to 28 GW.

Strengths of the present study include its population-based longitudinal design, the high attendance rate with minor loss to follow-up, and the relatively large sample size in a multiethnic European context. We also performed universal screening for GDM in 28 GW and assessed GDM by two definitions. A broad data set was collected that made us able to explore relations between 25(OH)D and several measures of glucose metabolism, and we adjusted for a range of possible confounders after drawing a DAG. Fore ethical reasons, women with 25(OH)D < 37 nmol/L were recommended vitamin D supplementation. As the main exposure variable 25(OH)D was measured at two time points in pregnancy, we were able to describe vitamin D levels during the first two trimesters (categories), where those who were recommended supplements can be followed. We measured 25(OH)D with standardized methods at the same high-quality laboratory, and half of the sample had vitamin D deficiency, many with severe deficiency.

The main limitation of our study is that the “gold standard” methods to quantify insulin resistance and *β*-cell function were not feasible in our primary care setting. The HOMA indexes are surrogate measures of insulin resistance and *β*-cell function estimated from FPG and fasting C-peptide concentrations. However, since HOMA-B is calculated from fasting values only, and the response over time after a glucose load, it has limited ability to detect chronic *β*-cell dysfunction, but HOMA is feasible in large studies and has been validated in pregnancy [28]. In addition, method-related differences in the measurement of 25(OH)D are widespread, although results from the Hormone Laboratory were found reliable compared with the gold standard of measuring 25(OH)D (standardized liquid chromatography-tandem mass spectrometry) [29]. Another limitation is that the categorisation of 25(OH)D may not be optimal as we do not separate women with a large increase in 25(OH)D from those with only a small increase from just below to above 37 nmol/L. Only very few women had levels above 100 nmol/L, and nearly all are from Europe, making comparison with 25(OH)D ≥ 100 nmol/L as reference impossible [30].

First, comparing studies may be hampered by different criteria of GDM, different definitions of vitamin D deficiency, and different methods of 25(OH)D measurements, and some studies have very low prevalence of vitamin D deficiency among the women included [7, 8]. We identified no other study exploring 25(OH)D at several time points during pregnancy in relation to GDM. In contrast to our population-based cohort study, several other observational studies have found an association between 25(OH)D and GDM [7, 8]. These studies are for the most part either cross-sectional or case-control/nested case-control studies, some are small, and some may represent selected groups. Another important issue in observational studies is adjustment for confounders. While most studies adjusted for age and BMI and some for season of blood drawn, some studies did not adjust for any confounders [10, 11]. In line with our study, some other studies did not find an association after adjusting for confounders [7, 8]. We used 50 nmol/L as the cutoff point for 25(OH)D, as this is the most used in literature. However, during pregnancy the conversion of 25(OH)D to 1,25(OH)_2_D may be optimized at higher levels (>100 nmol/L) [30]. Some studies report that 25(OH)D < 100 nmol/L was associated with adverse pregnancy outcomes, such as preeclampsia and preterm birth [31–33]. In our study, we were not able to analyse this relation as very few women had high levels, making comparison with 25(OH)D > 100 nmol/L as a reference impossible. However, linear regression analyses did not indicate any nonlinear relationships between 25(OH)D and measures of glucose metabolism, so our results do not suggest any specific cutoff points. If the variation of serum levels of 25(OH)D is small, it might be difficult to find an association with GDM. A strength of our study is a large range in serum levels and, in particular, many women with low values, in contrast to some other studies. To account for the range in 25(OH)D concentrations, we also analysed 25(OH)D as a continuous variable in regression models. Importantly, 25(OH)D was not significantly associated with GDM or other measures of glucose metabolism in these analyses. However, as mentioned, there were relatively few women with high levels (>100 nmol/L), which may have limited our possibility to assess these associations across the full range of 25(OH)D levels.

An association between vitamin D and body fat has been found in several studies [34, 35]. As body fat and weight gain are risk factors for GDM [18, 36], it is important to adjust for body fat as a confounder of the association between vitamin D and GDM. Asians are known to have a body composition with more visceral and central fat and more fat per BMI unit compared with Western subjects which contribute to an increased insulin resistance, particularly seen in South Asians [37]. Therefore, BMI is not a good measure of body fat across populations. Many of the studies have adjusted for BMI or prepregnant BMI, although BMI is affected by physiological changes during pregnancy, with increased body fluid, and weight of the foetus and placenta. In our study in a pregnant population, we measured the sum of skinfolds in addition to BMI, in early pregnancy and at the time of GDM diagnosis. In our primary analyses, we adjusted for sum of skinfolds and change in sum of skinfolds accounting for increasing fat deposits from inclusion to 28 GW by including change in sum of skinfolds [8]. We identified only two studies adjusting for weight gain or other measures of increasing fat deposits during pregnancy [38, 39]. In the sensitivity analysis, adjusting for prepregnant BMI and weight gain, vitamin D deficiency and GDM were still significantly associated. However, when adjusting for a more direct measure of fat deposits, the association was attenuated and no longer significant, probably reflecting that sum of skinfolds is a better measure of fat deposits in multiethnic pregnant populations. Furthermore, of the studies that found an association, only a few studies adjusted for socioeconomic status and demographics such as ethnicity. Many of these are studies from the USA primarily representing groups with African American and Hispanic ethnicities in addition to European-origin populations [7]. Studies from other countries representing other populations provide divergent results as one study from Iran [40] and one study from China [41] found an association, while a study from India did not [42].

Studies exploring relations between 25(OH)D and other measures of glucose metabolism are fewer, results are inconsistent, and studies are often hampered with methodological flaws, as few used a longitudinal design and adjustment for confounding factors. Many studies report only correlations, primarily with FPG, insulin, and different measures of HOMA, most showing inverse correlations with 25(OH)D. In studies adjusting for confounders, results are inconsistent [38, 40, 43–46].

The most striking finding in our study was that the association with 25(OH)D disappeared after adjusting for ethnicity both for the primary and secondary outcomes. We can only speculate about the reason for this. Ethnicity obviously reflects numerous factors; some may be related to vitamin D and some to GDM, but not necessarily to both. For example, skin pigmentation and use of concealing clothes and minimal sun exposure of the skin are associated with some ethnic groups and vitamin D status, but not necessarily with GDM, although these factors may be related to low levels of physical activity. Lifestyle factors, such as a low fibre-high simple carbohydrate diet and a low physical activity level, are strongly related to ethnicity and GDM, but not necessarily vitamin D. We have also found a strong relation with early life factors and GDM explaining some of the excess susceptibility for GDM in ethnic minority groups [47], but the relation with these factors and 25(OH)D is less clear. Further, genes involved in vitamin D metabolism or GDM, but probably not the same genes, could be differentially expressed depending on ethnic origin. Hence, ethnicity is an important confounder, but the effects on the exposure and the outcome are probably mediated through different mechanisms and could represent cultural, social, genetic, or other unmeasured factors.

Generally, most of the observational studies seem to be hampered by poor methodological design and probably residual confounding. Two reviews of trials concluded that vitamin D supplementation did not influence the incidence of GDM, but few and very small trials were found [48, 49]. Well-designed randomized controlled trials in multiethnic populations with low vitamin D status and high risk of GDM are considered necessary to determine the effect of vitamin D supplementation on prevention of GDM.

## 5. Conclusion

Vitamin D deficiency was not associated with GDM or glucose metabolism in a multiethnic population-based study, after adjustments for confounding factors. Our findings indicate that confounding by fat deposits and ethnicity explained most of the observed associations in unadjusted analysis.

## Figures and Tables

**Table 1:** 25-Hydroxyvitamin D (25(OH)D) status and confounding variables in the total sample and stratified by gestational diabetes mellitus (GDM) status, WHO 2013 criteria. Values are mean (95% confidence interval) or (numbers (%)).

		GDM (WHO 2013)		*p*
		Yes	No	
	*n* = 745	*n* = 235	*n* = 510	
*Status at inclusion (15 GW^i^)*				
Overall mean 25(OH)D (nmol/L)	50.2 (48.3, 52.1)	47.7 (44.0, 51.3)	51.4 (49.2, 53.7)	0.07
25(OH)D < 50 nmol/L (*n* (%))	389 (52)	141 (60)	248 (49)	**0.01**
*25(OH)D status during pregnancy^a^*				
Consistently sufficient (*n* (%))	417 (57)	109 (47)	308 (61)	**<0.01**
Decreasing (*n* (%))	63 (8.6)	22 (9.6)	41 (8.1)	0.50
Increasing (*n* (%))	145 (20)	55 (24)	90 (18)	0.06
Consistently deficient (*n* (%))	111 (15)	44 (19)	67 (13)	**0.03**
*Prepregnancy maternal status*				
Age (years)	29.8 (29.5, 30.2)	30.3 (29.6, 30.9)	29.6 (29.2, 30.1)	0.12
Ethnicity (*n* (%))				
Europe	346 (46.4)	82 (35)	264 (52)	**<0.01**
South Asia	191 (25.6)	81 (35)	110 (22)	**<0.01**
Middle East and North Africa	115 (15.4)	41 (17)	74 (15)	0.49
Sub-Saharan Africa	53 (7.1)	20 (8.5)	33 (6.5)	0.32
East Asia	40 (5.4)	11 (4.7)	29 (5.7)	0.57
Parity (*n* (%))				
Para 0	340 (46)	104 (44)	236 (46)	0.61
Para 1	256 (34)	73 (31)	183 (36)	0.18
Para ≥ 2	149 (20)	58 (25)	91 (18)	**0.03**
Education (years) (*n* (%))^a^				
<10	122 (16)	50 (21)	72 (14)	**0.02**
10–12	293 (40)	100 (43)	193 (38)	0.20
>12	324 (44)	84 (36)	240 (48)	**<0.01**
Prepregnancy BMI^ii^ (kg/m^2^)^a^	24.5 (24.2, 24.9)	25.9 (25.2, 26.6)	23.9 (23.5, 24.3)	**<0.01**
*Status at inclusion*				
Gestational week	15.1 (14.9, 15.4)	15.2 (14.7, 15.6)	15.1 (14.8, 15.4)	0.74
Sum of skinfolds (mm)^b^	72.0 (70.6, 73.5)	77.0 (74.3, 79.8)	69.8 (68.1, 71.5)	**<0.01**
Season for 25(OH)D measurement (*n* (%))				
Summer	347 (47)	130 (55)	217 (43)	**<0.01**
Winter	398 (53)	105 (45)	293 (57)	**<0.01**
*Status at 28 GW*				
Gestational week^c^	28.3 (28.2, 28.4)	28.2 (28.0, 28.3)	28.3 (28.2, 28.4)	0.20
ΔSum of skinfolds (15 to 28 GW) (mm)^d^	5.8 (4.8, 6.8)	6.2 (4.5, 7.9)	5.6 (4.3, 6.8)	0.58
Weight gain (prepregnant to 28 GW) (kg)	8.7 (8.3, 9.0)	8.8 (8.1, 9.4)	8.6 (8.2, 9.0)	0.71
Dietary clusters (*n* (%))^a^				
Healthy	239 (33)	54 (23)	185 (37)	**<0.01**
Unhealthy	493 (67)	179 (77)	314 (63)	**<0.01**

^i^GW: gestational week derived from the 1st day of the woman's last menstrual period; ^ii^BMI: body mass index; ^a^missing information of 5–13 women; ^b^ *n* = 681; ^c^missing information of 1–4 women; ^d^ *n* = 649. Consistently sufficient: 25(OH)D ≥ 37 nmol/L at 15 and 28 GW. Decreasing: 25(OH)D ≥ 37 nmol/L at 15 GW and <37 nmol/L at 28 GW. Increasing: 25(OH)D < 37 nmol/L at 15 GW and ≥37 nmol/L at 28 GW. Consistently deficient: 25(OH)D < 37 nmol/L at 15 and 28 GW. *p* values for the differences between GDM and non-GDM. Bold numbers indicate *p* values < 0.05. Independent *t*-test or two-sample test of proportions.

**Table 2:** Univariate and multiple regressions between 25-hydroxyvitamin D (25(OH)D) and gestational diabetes mellitus (GDM)^a^ (odds ratios and 95% confidence intervals). Associations according to vitamin D deficiency at inclusion (25(OH)D < 50 nmol/L) and vitamin D status during pregnancy (consistently sufficient or deficient, increasing or decreasing).

	Univariate analysis				Multiple analysis (model 1)			Multiple analysis (model 2)			Multiple analysis (model 3)		
	*n*	OR	(95% CI)	*p*	OR	(95% CI)	*p*	OR	(95% CI)	*p*	OR	(95% CI)	*p*
					*n* = 739, *R* ^2^ = 0.064			*n* = 645, *R* ^2^ = 0.113			*n* = 645, *R* ^2^ = 0.130		
25(OH)D sufficiency (≥50 nmol/L) at inclusion (15 GW) (ref)	745												
25(OH)D < 50 nmol/L		1.6	(1.2, 2.2)	**<0.01**	1.6	(1.1, 2.2)	**<0.01**	1.4	(0.95, 2.0)	0.09	1.1	(0.69, 1.6)	0.79
					*n* = 730, *R* ^2^ = 0.065			*n* = 640, *R* ^2^ = 0.108			*n* = 640, *R* ^2^ = 0.127		
25(OH)D consistently sufficient (ref)	736												
Decreasing		1.5	(0.86, 2.7)	0.15	1.4	(0.77, 2.5)	0.29	1.3	(0.68, 2.4)	0.49	1.1	(0.60, 2.1)	0.69
Increasing		1.7	(1.2, 2.6)	**<0.01**	1.8	(1.2, 2.7)	**<0.01**	1.4	(0.87, 2.2)	0.18	1.0	(0.60, 1.7)	0.97
Consistently deficient		1.9	(1.2, 2.9)	**<0.01**	1.7	(1.0, 2.7)	**0.04**	1.3	(0.74, 2.2)	0.39	0.88	(0.49, 1.6)	0.68

^a^Logistic regression analysis with GDM as dependent variable. ref: referent value; GW: gestational week; *R* ^2^: coefficient of determination. Model 1: adjusted for age, parity, education, and season. Model 2: the same as model 1, with additional adjustment for the sum of skinfolds at visit 1 and change in skinfolds from visit 1 to visit 2. Model 3: the same as model 2, with additional adjustment for ethnicity/geographic origin. Consistently sufficient: 25(OH)D ≥ 37 nmol/L at 15 and 28 GW. Decreasing: 25(OH)D ≥ 37 nmol/L at 15 GW and <37 nmol/L at 28 GW. Increasing: 25(OH)D < 37 nmol/L at 15 GW and ≥37 nmol/L at 28 GW. Consistently deficient: 25(OH)D < 37 nmol/L at 15 and 28 GW.

**Table 3:** Separate generalised linear models between 25-hydroxyvitamin D (25(OH)D) and each of the following dependent variables: fasting plasma glucose (FPG), HOMA-IR, fasting insulin, and C-peptide (regression coefficients and 95% confidence intervals). Associations according to vitamin D deficiency at inclusion (25(OH)D < 50 nmol/L) with vitamin D sufficiency (25(OH)D ≥ 50 nmol/L) as reference.

	Univariate analysis				Multiple analysis (model 1)			Multiple analysis (model 2)			Multiple analysis (model 3)^a^		
	*n*	*B*	(95% CI)	*p*	*B*	(95% CI)	*p*	*B*	(95% CI)	*p*	*B*	(95% CI)	*p*
FPG^a^	745	0.13	(0.04, 0.21)	**<0.01**	0.13	(0.04, 0.22)	**<0.01**	0.10	(0.01, 0.20)	**0.04**	0.017	(−0.10, 0.13)	0.77
HOMA-IR^b^	731	0.16	(0.04, 0.28)	**<0.01**	0.19	(0.07, 0.32)	**<0.01**	0.14	(0.01, 0.27)	**0.03**	0.045	(−0.10, 0.20)	0.54
Insulin^c^ (fasting)	731	16	(6.4, 25)	**<0.01**	15.2	(5.6, 25)	**<0.01**	10	(0.39, 20)	**0.04**	−0.13	(−11, 11)	0.98
C-peptide^d^ (fasting)	731	70	(14, 125)	**0.01**	84	(26, 141)	**<0.01**	63	(5.2, 122)	**0.03**	21	(−45, 87)	0.54

Model 1: adjusted for age, parity, education, and season. Model 2: the same as model 1, with additional adjustment for the sum of skinfolds at visit 1 and change in skinfolds from visit 1 to visit 2. Model 3: the same as model 2, with additional adjustment for ethnicity/geographic origin. ^a^FPG: *n* = 645, AIC (Akaike' information criterion) = 1182; ^b^HOMA-IR: *n* = 635, AIC = 1482; ^c^Insulin: *n* = 635, AIC = 6971; ^d^C-peptide: *n* = 635, AIC = 9259.

## Abbreviations

25(OH)D: 25-Hydroxyvitamin D
BMI: Body mass index
FPG: Fasting plasma glucose
DAG: Directed acyclic graph
GDM: Gestational diabetes mellitus
GW: Gestational week
HOMA: Homeostasis model assessment
HOMA-IR: Homeostasis model assessment of insulin resistance
HOMA-B: Homeostasis model assessment of *β*-cell function
PG: Plasma glucose.

## Supplementary Materials

Supplementary 1 Supplementary Table 1: characteristics of the cohort by geographic origin. Values are mean (standard deviation), numbers (%), or median (interquartile range (IQR)).

Supplementary 2 Supplementary Table 2: 25-hydroxyvitamin D (25(OH)D) status in the total sample and stratified by gestational diabetes mellitus (GDM) status, WHO 2013 criteria. Values are mean (95% confidence interval) or numbers (%).

Supplementary 3 Supplementary Table 3: Spearman's rank correlation coefficients between 25-hydroxyvitamin D (25(OH)D) at inclusion (15 GW) and measures of glucose metabolism at 28 GW.

Supplementary 4 Supplementary Figure 1: flow chart.
