# Supplementary material for: Vitamin D, Gestational Diabetes, and Measures of Glucose Metabolism in a Population-Based Multiethnic Cohort
Source: J Diabetes Res. 2018 Apr 19;2018:8939235. doi: 10.1155/2018/8939235 (PMC5933024; doi:10.1155/2018/8939235)
Supplement: Supplementary 1 — Supplementary Table 1: characteristics of the cohort by geographic origin. Values are mean (standard deviation), numbers (%), or median (interquartile range (IQR)). [file 8939235.f1.docx]

**Supplementary material**

**Supplementary Table 1.** Characteristics of the cohort by geographic origin. Values are mean (standard deviation), numbers (%) or median (interquartile range (IQR).

GW: gestational week. Gestational week derived from the 1st day of the woman's last menstrual period.

ᵅGDM: gestational diabetes mellitus. WHO (2013 criteria): fasting plasma glucose (FPG) ≥5.1 mmol/L or 2-hour plasma glucose (PG) ≥8.5 mmol/L.

^b^BMI: body mass index

^c^HOMA-IR: Homeostatic Model Assessment of Insulin Resistance

^d^HOMA-B: Homeostatic Model Assessment β-cell function

*Median with IQR.
