# Supplementary material for: Vitamin D, Gestational Diabetes, and Measures of Glucose Metabolism in a Population-Based Multiethnic Cohort
Source: J Diabetes Res. 2018 Apr 19;2018:8939235. doi: 10.1155/2018/8939235 (PMC5933024; doi:10.1155/2018/8939235)
Supplement: Supplementary 2 — Supplementary Table 2: 25-hydroxyvitamin D (25(OH)D) status in the total sample and stratified by gestational diabetes mellitus (GDM) status, WHO 2013 criteria. Values are mean (95% confidence interval) or numbers (%). [file 8939235.f2.docx]

**Supplementary Table 2**.

25-hydroxyvitamin D [25(OH)D] status in the total sample and stratified by gestational diabetes mellitus (GDM) status, WHO 2013 criteria. Values are mean (95% confidence interval) or [numbers (%)].

^i^GW: gestational week derived from the 1st day of the woman's last menstrual period.

P-values for the differences between GDM and non-GDM. Bold numbers indicate P-values <0.05.

Independent t-test or chi-square.
