# Supplementary material for: Vitamin D, Gestational Diabetes, and Measures of Glucose Metabolism in a Population-Based Multiethnic Cohort
Source: J Diabetes Res. 2018 Apr 19;2018:8939235. doi: 10.1155/2018/8939235 (PMC5933024; doi:10.1155/2018/8939235)
Supplement: Supplementary 3 — Supplementary Table 3: Spearman's rank correlation coefficients between 25-hydroxyvitamin D (25(OH)D) at inclusion (15 GW) and measures of glucose metabolism at 28 GW. [file 8939235.f3.docx]

**Supplementary Table 3.**

Spearman's rank correlation coefficients between 25-hydroxyvitamin D [25(OH)D]

at inclusion (15 GW) and measures of glucose metabolism at 28 GW.

n=697

GW: gestational week

ᵅHOMA-IR: Homeostatic Model Assessment of Insulin Resistance

^b^HOMA-B: Homeostatic Model Assessment of β-cell function

^c^FPG: fasting plasma glucose

Bold numbers indicate P-values <0.05.
