# Supplementary figures and images for: Vitamin D, Gestational Diabetes, and Measures of Glucose Metabolism in a Population-Based Multiethnic Cohort
Source: J Diabetes Res. 2018 Apr 19;2018:8939235. doi: 10.1155/2018/8939235 (PMC5933024; doi:10.1155/2018/8939235)

**Supplementary Figure 1. Flow chart.**


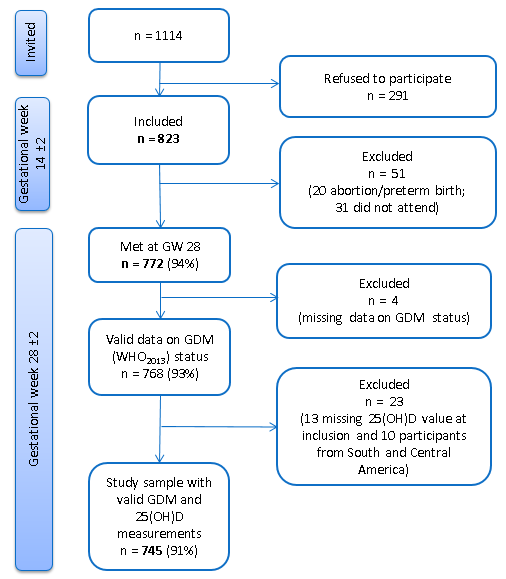

Supplement: Supplementary 4 — Supplementary Figure 1: flow chart. [file 8939235.f4.docx]
